# Multifunctional Optical Thin Films Fabricated by the Photopolymerization of Uniaxially Oriented Lyotropic Liquid Crystal Monomers for Electro-Optical Devices

**DOI:** 10.1038/srep36472

**Published:** 2016-11-04

**Authors:** Pureun Im, Yu-Jin Choi, Won-Jin Yoon, Dong-Gue Kang, Minwook Park, Dae-Yoon Kim, Cheul-Ro Lee, Seungbin Yang, Ji-Hoon Lee, Kwang-Un Jeong

**Affiliations:** 1 BK21 Plus Haptic Polymer Composite Research Team & Department of Polymer-Nano Science and Technology, Chonbuk National University, Jeonju, 54896, Republic of Korea; 2 Division of Advanced Materials Engineering, Chonbuk National University, Jeonju, 54896, Republic of Korea; 3 Division of Electronics Engineering, Chonbuk National University, Jeonju, 54896, Republic of Korea

## Abstract

A multifunctional optical thin film (MOTF) is fabricated by coating the newly synthesized perylene-based reactive mesogen (PBRM) and stabilized by the subsequent photopolymerization. Based on the spectroscopic results combined with morphological observations, it is found that nematic liquid crystal (NLC) is aligned parallel to the molecular long axis of PBRM not only due to the long-range physical anchoring effect but also due to the short-range molecular physical interactions between alignment layer and NLC molecules. From the electro-optical properties of LC test cells fabricated with the PBRM MOTF, it is clearly demonstrated that the PBRM MOTF can work as the planar LC alignment layer as well as the in-cell coatable polarizer. The coatable PBRM MOTF from lyotropic chromonic reactive mesogens can pave a new way for the flexible optoelectronic devices.

The control of molecular orientation is one of the most important technologies in the manufacturing process of liquid crystal device (LCD)^1^,^2^,^3^,^4^,^5^,^6^,^7^. Among various molecular alignment layers, the rubbed polyimide (PI) thin films have been commonly used as LC alignment layers because of their excellent thermal, chemical and mechanical stabilities as well as strong anchoring interactions with LC molecules^8^,^9^,^10^,^11^,^12^. However, the mechanical contact method often generates various unfavorable particles, such as debris, dust particles, and unstable static charges, which can induce adverse effects to the electrical and optical performances of LCD devices^13^,^14^,^15^,^16^. In addition, high-molecular weight polymers possess high viscosity which leads to long annealing times at high temperatures with toxic solvents^17^. To overcome the drawbacks of traditional alignment layers, many alternative methods including photo-alignment layers and self-assembled alignment layers have been introduced^18^,^19^,^20^,^21^,^22^. In parallel to the development of alignment layer, the coatable multifunctional optical thin films wherein the single film functions both as the alignment layer and polarizer have been intensively required from optoelectronic device engineers for the fabrication of flexible displays and devices^23^,^24^,^25^,^26^,^27^.

Recently, our research group successfully fabricated the coatable thin film polarizer by the polymer-stabilization of macroscopically oriented lyotropic chromonic liquid crystal (LCLC)^28^,^29^,^30^,^31^. The LCLC-based coatable polarizers stabilized by the photopolymerization of LCLC monomers exhibit high polarizabilities as well as excellent chemical and mechanical resistances. It is worth noticing that the LCLC-based coatable thin film is a E-type polarizer which transmits the extraordinary ray and extincts the ordinary ray, while the conventional polarizer using the stretched-polymer is a O-type polarizer which transmits the extraordinary ray and extincts the ordinary ray. Thus, the incident light polarized parallel to the long molecular axis of the LCLC molecule can transmit the polarizer^29^,^30^,^31^,^32^,^33^,^34^,^35^.

In this paper, our research group introduces a multifunctional optical thin film (MOTF) that can simultaneously serve as an in-cell polarizer as well as a planar alignment layer for nematic LC (NLC). To realize this concept, a perylene-based reactive mesogenic molecule (abbreviated as PBRM) containing diacrylate functional groups at the ends of the molecule is strategically designed and synthesized^36^,^37^,^38^. The PBRM MOTF is fabricated by the simple shear coating and photopolymerization process. Their optical property and quantitative polarizability are observed by employing the polarized optical microscopy (POM) and polarized ultraviolet-visible (UV-vis) spectroscopy. To investigate the ability of the PBRM MOTF as a LC alignment layer, various LC test cells are fabricated and filled with a pentyl-cyanobiphenyl (5CB) nematic liquid crystal (NLC). The molecular alignment behaviors of the 5CB molecules on the PBRM MOTF layer are estimated by observing the LC test cells under POM and polarized FT-IR spectroscopy. Furthermore, the pretilt angle and polar anchoring energy are measured to identify the molecular physical interactions between the 5CB molecules and the PBRM MOTF layer. Electro-optical switching properties of the 5CB molecules in the twist nematic (TN) as well as in the in-plane switching (IPS) LC test cells are investigated wherein the PBRM MOTF is used as the alignment layer.

## Results and Discussion

### Anisotropic optical property of the perylene-based reactive mesogen

**Figure 1:**
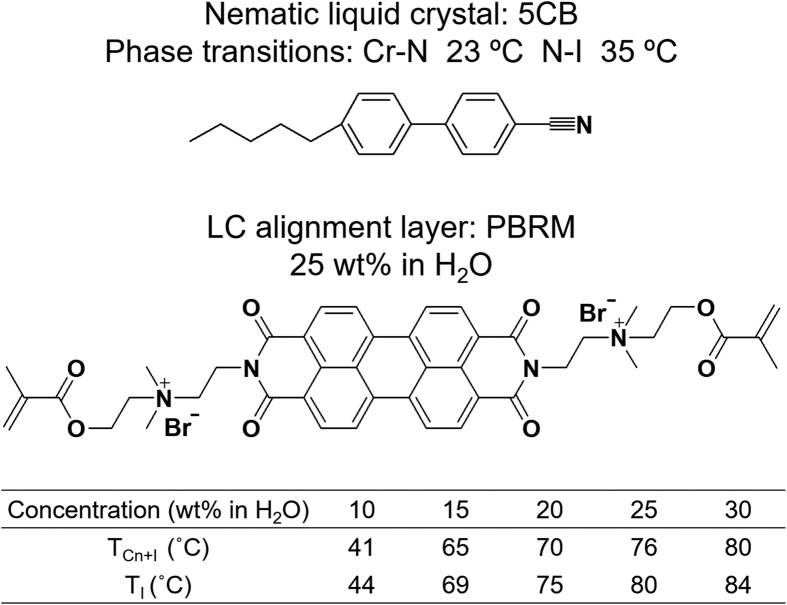
Anisotropic optical molecules. Materials information: nematic liquid crystal (NLC, 5CB) with its phase transition temperatures, and PBRM with the phase transitions of PBRM-H_2_O solution.

For the fabrication of multifunctional optical thin film (MOTF) which can be applied as an internal polarizer as well as an alignment layer, a perylene-based reactive mesogen (PBRM) is synthesized and the detail synthetic procedures are represented (see Supplementary Fig. S1) and found in the refs 36, 37, 38. The acrylation reaction between anhydride and alcohol in the presence of 4-dimethylaminopyridine (DMAP) results in the targeted PBRM molecule. High yield and purity are confirmed by the proton (^1^H) nuclear magnetic resonance (NMR) spectroscopy and electrospray ionization mass spectroscopy (ESI-MS). The concentration of the PBRM in aqueous solutions is varied between 25 and 75 wt% in the presence of the 1 wt% water-soluble photo-initiator^31^. The PBRM MOTF is simply fabricated by the mechanical shear coating on the indium-tin oxide (ITO)-coated glass and the subsequent photopolymerization (see Supplementary Fig. S2). The materials information and optical properties of NLC and PBRM are shown in Fig. 1. The 5CB (Aldrich) NLC showing nematic phase at room temperature is used for the investigation of the alignment and the electro-optical properties of the sample fabricated with the PBRM MOTF. It is well known that 5CB molecules are uniaxially aligned on the PI planar alignment layer.

The phase transition of PBRM-H_2_O with respect to temperature and concentrations was fully estimated in our previous work^31^ and it is summarized in Fig. 1. A typical columnar nematic (Col_N_) phase forms between 10 to 30 wt% at room temperature. At concentrations over 30 wt%, on the other hand, the crystallized particles are precipitated out of the aqueous solution. Upon increasing the temperature of PBRM-H_2_O solutions between 10 and 30 wt%, the Col_N_ phase transforms to the I phase by passing the biphase (Col_N_ + I) region. It is worth noticing that the formation of biphase between Col_N_ and I phases is one of the typical characteristics of LCLC compounds^31^. For the fabrication of macroscopically oriented PBRM MOTF, the concentration of PBRM solution is fixed to be 25 wt%, which is optimized based on the results of POM morphological observations and degree of polarization (DOP).

**Figure 2:**
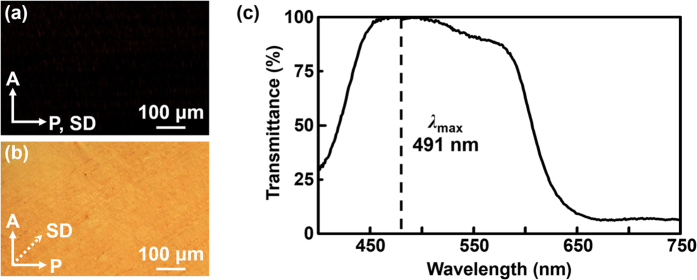
Coatable polarizer. POM images of the uniaxially oriented photopolymerized PBRM MOTF when the angle between SD and polarizer is (**a**) parallel and (**b**) 45°, respectively. (**c**) Degree of polarization (DOP) of the PBRM MOTF which is calculated from the corresponding polarized UV-vis spectrum.

The optical property and morphology of fabricated PBRM MOTF are investigated by linear cross-polarized optical microscope (POM). As shown in Fig. 2(a,b), the uniaxially oriented PBRM MOTF shows the optical property of E-type polarizer. When the shear direction (SD) of PBRM MOTF is aligned parallel to the polarizer axis (P), the incident light is almost blocked by the analyzer (A) as shown in Fig. 2(a). The amount of transmitted light is increased by rotating the SD and the maximum transmittance is achieved when SD makes 45° to the polarizer in the Fig. 2(b). Additionally, any macroscopic cracks or defects are undetectable by morphological analysis, which indicate that the molecular arrangements are well maintained even after the overall fabrication processes. The degree of polarization (DOP) is quantitatively measured from the polarized UV-vis spectra obtained under the transmittance mode by rotating the UV-vis polarizer, as represented in Fig. 2(c). The DOP can be calculated from the following equation; DOP = (T_0_ − T_90_)/(T_0_ + T_90_)^29^,^39^,^40^. Here, T_0_ and T_90_ mean the transmittances when the azimuthal angles are 0° and 90° to the polarization axis of the UV-vis polarizer, respectively. The measured DOP of PBRM MOTF is determined to be 99.81% at *λ*_max_ = 491 nm after the normalization.

### Alignment behavior of the 5CB on the PBRM MOTF

**Figure 3:**
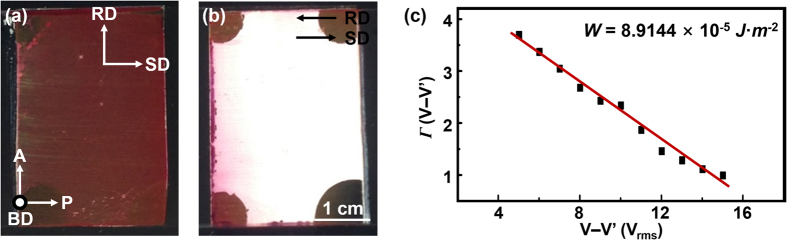
Surface alignment layer. Fabricated LC test cells with PBRM MOTF. Macroscopic images of the LC cells when the RD and SD are (**a**) perpendicular and (**b**) parallel to each other, respectively. (**c**) Surface anchoring energy (W) evaluated from the linear relationship of Г (V − V’) with respect to (V − V’).

To confirm the ability of PBRM MOTF as a LC alignment layer, different LC test cells are fabricated in two ways by sandwiching of PI and PBRM MOFT-coated ITO glass substrate; one is that the rubbing direction (RD) of PI layer and the SD of PBRM MOTF are assembled antiparallel and the other is that the RD and the SD are perpendicular to each other. It is worth noticing that the applied PI alignment layer induces the planar alignment of NLC molecules along the RD^11^,^12^,^41^. The NLC molecules are injected into the fabricated LC test cells by capillary action at 40 °C (isotropic phase of 5CB molecules). The flow effects which can be generated during the injection process are eliminated by slowly cooling down the cells to room temperature. The surface molecular alignment of the 5CB is investigated under cross-polarizers using the unpolarized light source. As shown in Fig. 3(a), when SD and RD are perpendicular each other, the light is blocked by the polarizer. On the other hand, the maximum transmittance is achieved by rotating SD about 45° to the polarizer. This result is similar to the LC orientation of the electrically controllable birefringence (ECB) mode wherein the PI-coated substrates are antiparallel rubbed^42^,^43^. When the RD is antiparallel to the SD, the high value of transmittance is obtained which can be shown a normally white state of the twisted nematic (TN) mode under cross-polarizers as represented Fig. 3(b)^13^,^44^. From these experimental results, it is concluded that the long-axis of 5CB is aligned perpendicular to the SD. We should note that the PBRM columns are aligned along the SD, while the long axis of the PBRM molecules are aligned perpendicular to the SD. Therefore, the 5CB molecules are aligned parallel to the PBRM molecule during the coating process^31^.

To examine the physical interactions between PBRM MOTF substrate and 5CB molecules, the pretilt angle (*θ*) and surface anchoring energy (*W*) are investigated by the LC test cells where the PBRM MOTF-coated ITO substrates are antiparallel assembled. The properties of 5CB at 23 °C are as follows; *K*_1_ = 6.65 × 10^−12^ N, *K*_3_ = 8.95 × 10^−12^ N; *n*_e_ = 1.717, *n*_o_ = 1.530; *ϵ*_⊥_ = 8.0, *ϵ*_∥_ = 19.5^45^. Here, *K*_1_ and *K*_3_ stand for the splay and bend elastic constants; *n*_o_ and *n*_e_ are ordinary and extraordinary refractive indices; *ϵ*_⊥_ and *ϵ*_∥_ mean the components of the dielectric tensors which are parallel and perpendicular to the director, respectively. The pretilt angle (*θ*) is defined as the angle between the average direction of the LC directors and surface plane^46^,^47^. The *θ* of NLC on the alignment layer affects the electro-optical performances, such as response time, driving voltage, and viewing angle. The *θ* is measured by checking the phase retardation (*Г*) of the sample. The *Г* is defined as 2*π*Δ*n(θ*)*d/λ*, where the birefringence is Δ*n(θ*) − *n*_o_ = [cos^2^*θ n*_o_^−2^ + sin^2^*θ n*_e_^−2^]^−1/2^ − *n*_o,_ *d* is the cell gap, and *λ* is the wavelength of light of the probe beam^48^. To measure *Г* of the sample, a He-Ne laser (*λ* = 632.8 nm) is passed through a polarizer, a Soleil-Babinet compensator, the cell, an analyzer, and a detector. The polarizing axes of the polarizer and analyzer are at −45° and +45° to the SD, respectively. The cell gap (*d*) is controlled to be 7.8 μm with ball spacers. The *Г* is measured with increasing the applied voltage and simultaneously adjusting the Soleil-Babinet compensator. Because the PBRM MOTF has its own phase retardation, we first measure the phase retardation of the empty cell and subtract it from the phase retardation of the LC-filled sample. The measured *θ* was 0.1° which is significantly smaller than the conventional PI-coated LC cell (2–3°). The low *θ* property can be especially very useful for the realization of the symmetric viewing angle of the LCD panels.

Polar anchoring energy (*W*) indicating the energetic cost to deviate LC from the initial alignment state is determined by the high-electric field techniques^49^,^50^. As shown in Fig. 3(c), (*Г/Г*_o_ − 1)(*V* − *V’*) is plotted with respect to *V* − *V’*, where *Г* is the phase retardation under the given voltage, *Г*_o_ is the maximum retardation under 40 V, *V*′ = *σβV*_th_, where σ = 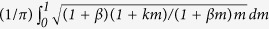 with *β* = (*ϵ*_⊥/_*ϵ*_∥_) − 1 and *k* = (*K*_11_/*K*_33_)−1. The slope of the graph corresponds to 2*K*_33_/*W*_d_. From the fitted experimental data, the *W* is determined to be 8.91 × 10^−5^ J m^−2^ at *R* = 0.28.

Polarized FT-IR spectroscopy is a commonly used technique to estimate the intra- and inter-molecular interactions without damaging the specimen^28^,^51^,^52^,^53^. The 5CB-filled LC cell was prepared by attaching two PBRM MOTF in antiparallel. Since 5CB molecule has a cyanide functional group (C≡N) at the end, the significant FT-IR absorption band corresponding to the stretching vibration of C≡N is detected at 2225 cm^−1^ (see Supplementary Fig. S3)^54^. The dichroism data are obtained by varying the polarization axis from 0° to 360° with respect to the SD of PBRM MOTF. Here, 0° indicates that the SD is parallel to the polarization axis of the IR polarizer.

**Figure 4:**
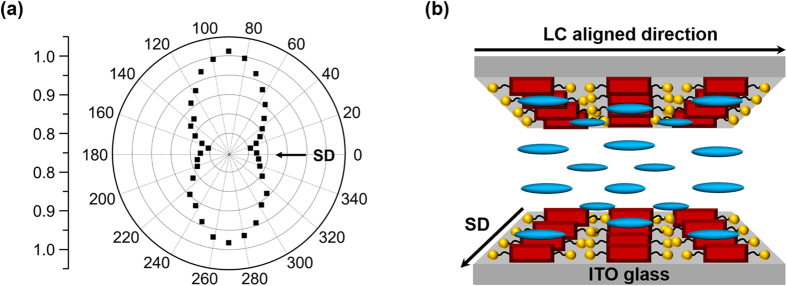
Molecular alignment on the MOTF. Polarized IR absorption intensity of the LC cell rotating the optic axis of polarizer. Here, 0 indicates that the SD of PBRM MOTF and the optic axis of IR polarizer are parallel to each other. (**b**) A schematic illustration of the aligned NLC molecules on the PBRM MOTF.

As summarized in Fig. 4(a), the weak absorption intensity is measured at 0° and 180°. With rotating the polarizer, the absorption is continually increased and the strong absorptions are detected at 90° and 270°. From this experimental result, it is conformed that the cyanide group predominantly aligns perpendicular to the UV polarization direction. In other words, the 5CB is arranged perpendicular to the SD of PBRM MOTF and the experimental result is well matched with those of test cells (Fig. 4(b)). It is a common knowledge that the orientations of NLC are controlled by the anisotropic surface of the PI alignment layer^10^,^11^,^16^. The origin of NLC alignment on PBRM MOTF is affected not only by the long-range physical anchoring, but also by the short-range molecular physical interactions between alignment layer and NLC molecules. The 5CB molecule consisting of in-line phenyl groups and polar cyanine group has a cylindrical symmetry along the rod direction. The polarity of PBRM MOTF is very high due to the carbonyl groups in the molecule and the absence of long alkyl chain. Surface polarity of alignment layer is rarely changed during the coating processes if the molecules containing few alkyl side groups. Note that a short side chain such as methyl and ethyl does not affect the direction of alignment and the pretilt angle^55^,^10^. On the other hand, the long side chains can push NLC molecules upward resulting in a relatively large pretilt angle. During the photopolymerization process, the highly oriented polar PBRM molecules form the polymer chains parallel to the self-assembled nanocolumn direction. It is generally known that molecular scale on the surface is more important than the macroscopic microgrooves at the macroscopic scale^11^. Furthermore, the polar groups of PBRM molecule such as carbonyl groups are oriented along the out-of-plane^10^. From the short-range molecular physical interactions, the stronger electronic interactions between carbonyl groups of the PBRM molecule and the cyanine groups of the 5CB molecule on the surface can induce the orientation of the 5CB molecules along the PBRM molecular direction and result in a small pretilt angle close to zero. Therefore, we successfully demonstrate that PBRM MOTF can act as a planar LC alignment layer as well as an in-cell coatable polarizer.

### Electro-optical switching properties of the TN and IPS LC test cells with the PBRM MOTF

Although the behaviors of 5CB molecule are identified at the zero electric field, the electro-optical switching properties of the LC test cells should be investigated for the practical optoelectronics application. The voltage-transmittance (V-T) curve is obtained under cross-polarizers as increasing the electric field. For the fabrication of TN and IPS test cells, the PBRM MOTF-coated ITO glass is used as a top substrate and the PI-coated ITO glass is used as a bottom substrate. The cell gaps of the TN and IPS cell are controlled to be 2.7 μm and 1.8 μm, respectively.

**Figure 5:**
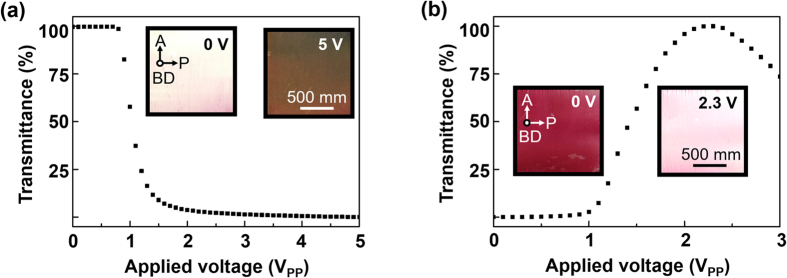
Electro-optical LC devices with MOTF. Electro-optical response of the (**a**) TN and (**b**) IPS LC test cells upon increasing the applied voltage and their corresponding POM images.

For the TN cell, the SD direction is parallel to the rubbing direction of PI. Because the polarization direction of light after passing the PBRM layer is along the SD and the 5CB molecules are oriented parallel to the polarizing direction, the PBRM MOTF-coated substrate can be used as a polarizer. Therefore, the probe light consecutively passes through PBRM MOTF-coated ITO glass (polarizer), 5CB layer, PI-coated ITO glass and analyzer. At the zero electric field, the fabricated TN cell displays a uniform white color throughout the whole area of the cell, which shows that the Mauguin’s condition generally called a normally white mode in the Fig. 5(a) 56,57. By increasing the applied voltage, the orientation of 5CB changes from the twisted to the homeotropic states with decreasing transmittance. The threshold voltage (V_th_) is experimentally identified to be 0.85 V. The inset images of Fig. 5(a) are macroscopic observations at 0 V and 5 V applied state, corresponding to the twisted and homeotropic states, respectively. Its electrical behavior coincides with typically observed V-T characteristics of commercial TN cell when the NLC aligns with the rubbed PI surface.

For the IPS mode cell, the SD direction is perpendicular to the rubbing direction of PI. In case of the IPS cell, the SD of PBRM MOTF is perpendicular to the interdigitated electrodes. The back light source is blocked under cross-polarizer of the IPS cell at the zero electric field. As the applied voltage exceeds the threshold voltage (V_th_), the transmittance gradually increases owing to the reorientation of the 5CB following the external electric field as shown in Fig. 5(b). The V_th_ of the IPS cell is measured to 1.126 V. Over the maximum brightness, the transmittance is reduced due to the retardation over the half of the wavelength of light. The macroscopic images correspond to the 0 V and 2.3 V applied state.

**Figure 6:**
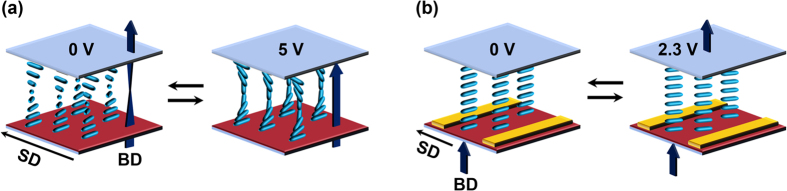
NLC molecular orientations in the LC test cells. Schematic illustrations of NLC molecular arrangement in the (**a**) TN and (**b**) IPS LC test cells according to the electrical fields.

Based on the experimental results, schematic illustrations of NLC molecular arrangement in the TN and IPS LC test cells according to the electrical field are represented in the Fig. 6. With increasing the applied voltage, the orientation of 5CB changes from the twisted to the homeotropic states, as schematically shown in Fig. 6(a). The 5CB molecule in the IPS LC test cells is rotated towards the direction of electric field as schematically provided in Fig. 6(b).

To observe the molecular behavior of the 5CB with and without PBRM MOTF, the patterned LC cell is also fabricated by selectively introducing photo-mask between the light source and the uniaxially coated PBRM MOTF. To be specific, the PBRM monomers in the open slit are polymerized with the UV irradiation while the blocked parts with the photo-mask are remained as monomers which can removed by the subsequent solvent development. The macroscopic images of the TN cell under cross-polarizer are shown (see Supplementary Fig. S4(a,b)) when the voltage is 0 V and 3 V, respectively. The 5CB molecules are well aligned on the PBRM MOTF but disordered on the developed region at the zero electric field. By applying the voltage, all the 5CB molecules move along the electric field direction and vertically stand regardless of patterned or not. To observe the morphology of the cell, the patterned region of cell with marking dashed circle is closely observed by using POM (see Supplementary Fig. S4(c,d)). Since 5CB molecules are randomly oriented, the transmittance of the cell is somewhat high at 0 V. Upon increasing the voltage, the transmittance is gradually decreased and the homeotropic state is obtained.

## Conclusions

A programmed perylene-based reactive mesogen (PBRM) is newly synthesized for the fabrication of multifunctional optical thin film (MOTF) which can work for the in-cell color polarizer as well as the planar LC alignment layer. The macroscopically oriented PBRM MOTF is stabilized by the UV photopolymerization. From the systematic experiments of PBRM MOTF, it is found that the 5CB molecule is aligned parallel to the PBRM molecule. The LC alignment on the PBRM MOTF is mainly affected by the short-range molecular physical interactions between the polar carbonyl groups and cyanine groups. The fabrication of the TN and IPS LC test cells are successfully demonstrated by applying PBRM MOTF and their electro-optical responses are also investigated. The PBRM MOTF fabricated by the simple shear coating and the subsequent photopolymerization can be the stepping stone for the flexible optoelectronic devices.

## Additional Information

**How to cite this article**: Im, P. *et al.* Multifunctional Optical Thin Films Fabricated by the Photopolymerization of Uniaxially Oriented Lyotropic Liquid Crystal Monomers for Electro-Optical Devices. *Sci. Rep.* **6**, 36472; doi: 10.1038/srep36472 (2016).

**Publisher’s note:** Springer Nature remains neutral with regard to jurisdictional claims in published maps and institutional affiliations.

## Supplementary Material

Supplementary Information
