# Supplementary material for: Multifunctional Optical Thin Films Fabricated by the Photopolymerization of Uniaxially Oriented Lyotropic Liquid Crystal Monomers for Electro-Optical Devices
Source: Sci Rep. 2016 Nov 4;6:36472. doi: 10.1038/srep36472 (PMC5095604; doi:10.1038/srep36472)
Supplement: Supplementary Information [file srep36472-s1.doc]

Supplementary Information

**Multifunctional Optical Thin Films Fabricated by the Photopolymerization of Uniaxially Oriented Lyotropic Liquid Crystal Monomers for Electro-Optical Devices**

Pureun Im,^†,⊥^ Yu-Jin Choi,^†,⊥^ Won-Jin Yoon,^†^ Dong-Gue Kang,^†^ Minwook Park,^†^ Dae-Yoon Kim,^†^ Cheul-Ro Lee,^‡^ Seungbin Yang,^§^ Ji-Hoon Lee,^§^,* and Kwang-Un Jeong^†^,*

^†^Polymer Materials Fusion Research Center & Department of Polymer-Nano Science and Technology, Chonbuk National University, Jeonju, 54896, Republic of Korea.

^‡^Division of Advanced Materials Engineering, Chonbuk National University, Jeonju, 54896, Republic of Korea.

^§^Division of Electronics Engineering, Chonbuk National University, Jeonju, 54896, Republic of Korea.

Correspondence and requests for materials should be addressed to J.H.L (E-mail: jihoonlee@jbnu.ac.kr) and K.U.J. (E-mail: kujeong@jbnu.ac.kr)

**Experimental method**

***Equipment and experiment:*** The surface morphology of the PBRM MOTF was studied by utilizing POM (Nikon ECLIPSE LV100POL). To determine the degree of polarization, polarized UV-vis spectroscope (Scinco S-3100) was obtained from the transmittances with respect to the angles between optic axes of UV-vis polarizer and PBRM MOTF. The wavelength of UV-vis polarizer was in the range of 250-3000 nm and the sample was coated and photo-polymerized on the quartz substrate. The polarized FT-IR spectra were obtained from Shimadzu IRTracer-100 with the IR polarizer. The electric responses corresponding to the electric field were evaluated by the LCMS-200. (Sesim Photonics Technology)

***Materials Preparation:*** Perylene-3,4,9,10-tetracarboxylic dianhydride (97%, Aldrich), N,N-dimethylethylene diamine (98%, TCI), 2-bromoethanol (95%, TCI), 4-(dimethylamino)pyridine (99%, Aldrich), methacrylic anhydride (94%, Aldrich) were used. All reactants and reagent materials for synthesis were purchased from Sigma Aldrich and TCI. These materials were used as received without further purifications. The PBRM and their derivatives (PDID-1, 2) were synthesized according to the literature and the synthetic procedure was represented in Fig. S1. Chemical structures and their purities were identified by the ^1^H NMR. The trifluoroacetic acid was used as a solvent for the characterization of the PDID-1 and deuterated water was also used for PDID-2 and PBRM.

***Synthesis of PDID-1:*** N,N-dimethylethylene diamine (4 ml, 20 mmol) was added into the perylene-3,4,9,10-tetracarboxylic dianhydride (2.5 g, 6.4 mmol) suspended in dried DMF (25 ml) and the solution was subsequently refluxed for 5 h at 130 °C. After the reaction, a suspension was annealed to room temperature and THF (150 ml) was added into the flask. The precipitate was filtered and washed with THF (300 ml). After drying in the vacuum oven for overnight, the resulting product (abbreviated as PDID-1) was obtained as a dark brown solid. (3.15 g, 92%); ^1^H NMR (400 MHz, TFA-d,): δ = 8.66 (d, 8H), 4.71 (t, 4H), 3.73 (d, 4H), 3.1 (s, 12H) ppm.

***Synthesis of PDID-2:*** A suspension of PDID-1 (1.5 g, 2.8 mmol) in 2-bromoethanol (1.6 ml, 22 mmol) was slowly heated to 100 °C and refluxed for 12 h. The solution was cooled down to room temperature and 100 ml of THF was added to obtain the precipitated product. The resulting precipitates were filtered with glass filter and washed with THF (450 ml). The glass filter with the product was dried in the vacuum oven for overnight and the crude product was dissolved in deionized water (100 ml). The solution was filtrated with a filter paper and subsequently freeze-dried for 3 days. The reddish solids were obtained. (2 g, 91%). ^1^H NMR (400 MHz, D_2_O): δ = 7.87(bs, 4H), 7.4 (bs, 4H), 4.5 (t, 4H), 4.0 (t, 4H), 3.7-3.8 (t, 8H), 3.18 (s, 12H) ppm.

***Synthesis of PBRM:*** PDID-2 (0.5 g, 0.6 mmol) and DMAP (20 mg, 0.2 mmol) were dissolved in the dried DMF (50 ml) and stirred for 15 min at room temperature. Methacrylic anhydride (1.0 ml, 6.4 mmol) was slowly added under light protection and stirred at 25 °C for 24 h. The crude product was poured into THF (200 ml) and the resulting precipitates were filtered and washed with THF (300ml). The final reddish product was obtained after drying in the vacuum oven (0.52 g, 89%). 1H NMR (400 MHz, D_2_O): δ = 7.9 (bs, 4H), 7.42 (bs, 4H), 6.53 (s, 2H), 6.18 (s, 2H), 5.15 (t, 4H), 4.9 (t, 4H), 4.58 (t, 4H), 4.11 (t, 4H), 3.77 (s, 12H), 2.3 (s, 6H) ppm. ESI-MS (m/z): [M]^2+^ calcd. for C_44_H_46_N_4_O_8_: 758.3; found, 758.6.

The fabrication of PBRM MOTF was schematically represented in Fig. S2. The composition of the DAPDI-H2O solution was optimized to be 25:75 with weight ratio with the 1% water-soluble photo-initiator (2-hydroxy-4’-(2-hydroxyethoxy)-2-methylpropiophenone, Sigma Aldrich). The solution was coated on the ITO substrates by the simple doctor blade coating methods by adjusting the height of the thickness to 3 μm and dried at 25 °C for 12 h. The macroscopically oriented PBRM layer was photo-polymerized for 30 min under the UV light with the intensity of 20 mW cm^-2^. The FT-IR spectra according to the angles between the fabricated cell and IR polarizer were obtained as shown in Fig. S3 and the absorption band at 2225 cm^-1^ corresponds to the stretching vibration of cyanide group of the 5CB.


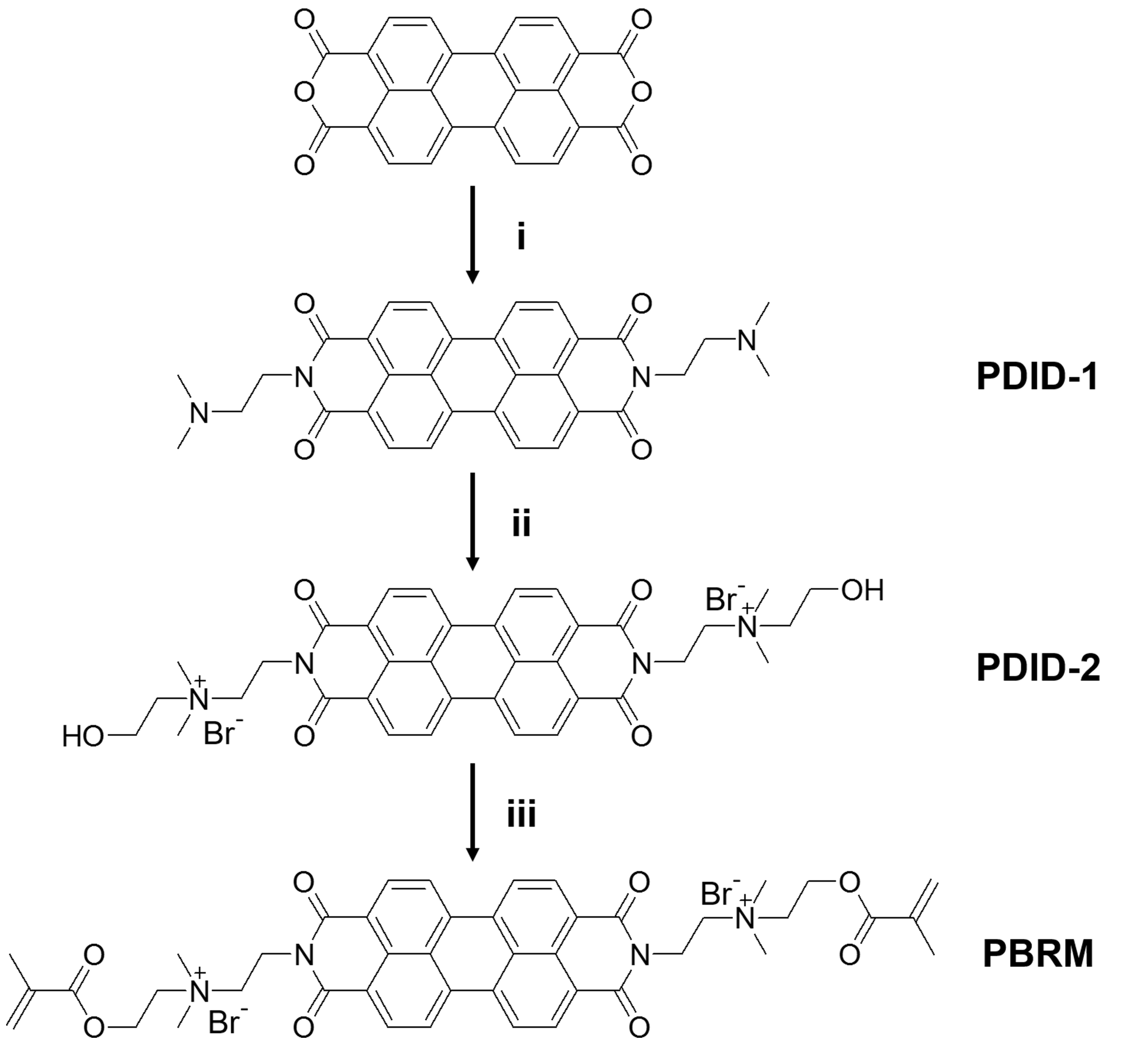


**Figure S1.** Synthetic procedures of PBRM and its intermediates, PDID-1 and PDID-2.

**
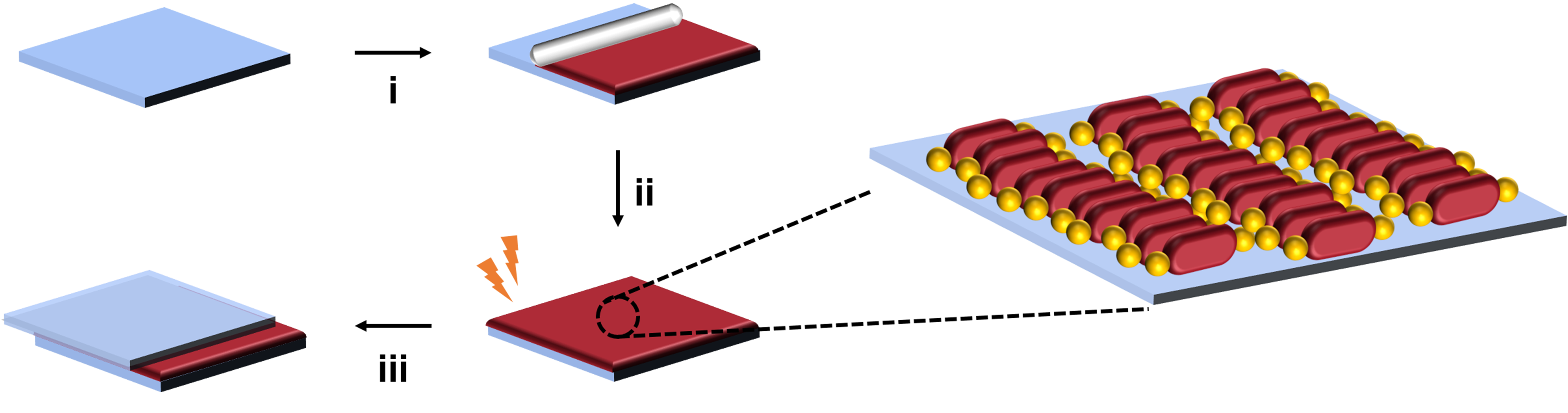
**

**Figure S2.** Fabrication of PBRM MOTF: i) the coating of the aqueous solution with 25 wt% PBRM on the ITO glass, ii) the photo-polymerization by irradiating the UV light (20 mW/cm^2^) for 30 min after the drying process for 12 h at RT, and iii) the fabrication of LC test cells by applying the PBRM MOTF.


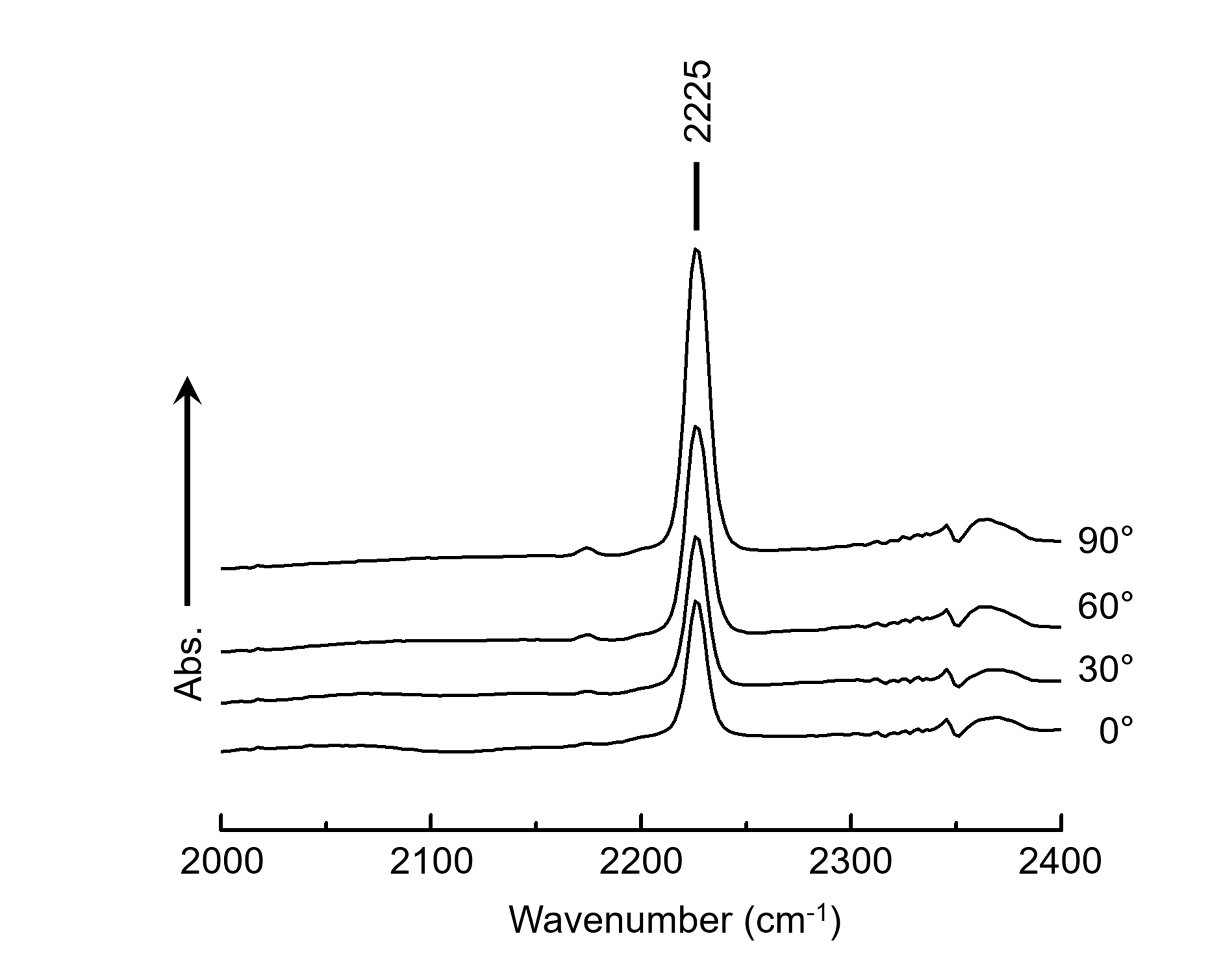


**Figure S3.** Polarized FT-IR spectra between 2000 to 2400 cm^-1^ obtained by changing the angle between the SD of PBRM MOTF and the optic axis of IR polarizer.


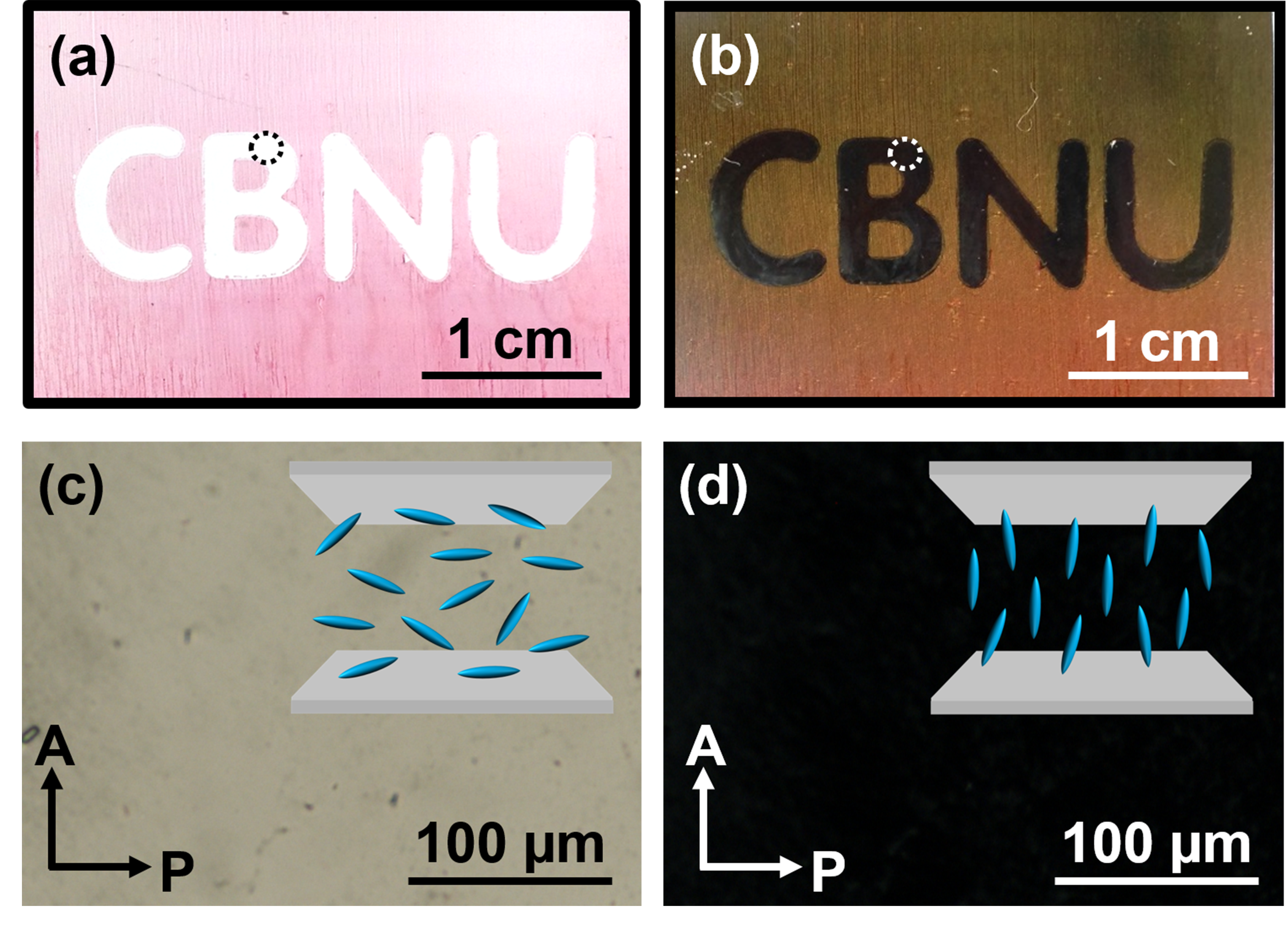


**Figure S4.** Macroscopic images of the TN LC test cells fabricated with the patterned PBRM MOTF at (a) 0 V and (b) 3 V, respectively. (c, d) POM images of the circled areas of the macroscopic images of the TN LC test cells (a, b), respectively.
